# Design and Synthesis of a Biotinylated Chemical Probe for Detecting the Molecular Targets of an Inhibitor of the Production of the *Pseudomonas aeruginosa* Virulence Factor Pyocyanin

**DOI:** 10.3390/molecules181011783

**Published:** 2013-09-25

**Authors:** Ysobel R. Baker, Warren R. J. D. Galloway, James T. Hodgkinson, David R. Spring

**Affiliations:** Department of Chemistry, University of Cambridge, Lensfield Road, Cambridge CB2 1EW, UK; E-Mails: yrb20@cam.ac.uk (Y.R.B.); wrjdg2@cam.ac.uk (W.R.J.D.G.); jth26@cam.ac.uk (J.T.H.)

**Keywords:** quorum sensing, *Pseudomonas aeruginosa*, anti-bacterial, target identification, virulence factor

## Abstract

*Pseudomonas aeruginosa* is a human pathogen associated with a variety of life-threatening nosocomial infections. This organism produces a range of virulence factors which actively cause damage to host tissues. One such virulence factor is pyocyanin, known to play a crucial role in the pathogenesis of *P. aeruginosa* infections. Previous studies had identified a novel compound capable of strongly inhibiting the production of pyocyanin. It was postulated that this inhibition results from modulation of an intercellular communication system termed quorum sensing, via direct binding of the compound with the LasR protein receptor. This raised the possibility that the compound could be an antagonist of quorum sensing in *P. aeruginosa*, which could have important implications as this intercellular signaling mechanism is known to regulate many additional facets of *P. aeruginosa* pathogenicity. However, there was no direct evidence for the binding of the active compound to LasR (or any other targets). Herein we describe the design and synthesis of a biotin-tagged version of the active compound. This could potentially be used as an affinity-based chemical probe to ascertain, in a direct fashion, the active compound’s macromolecular biological targets, and thus better delineate the mechanism by which it reduces the level of pyocyanin production.

## 1. Introduction

**Figure 1:**
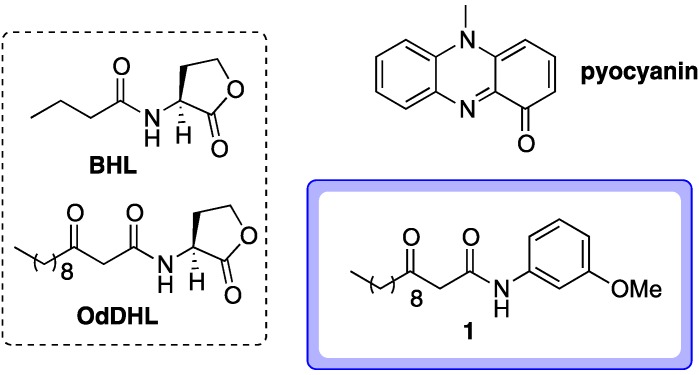
BHL and OdDHL are two natural AHL autoinducers used by *P. aeruginosa* in quorum sensing. Pyocyanin is a virulence factor produced by *P. aeruginosa*. Compound **1**, an abiotic OdDHL-mimic, is capable of strongly inhibiting the production of pyocyanin in cultures of wild type *P. aeruginosa* [1].

*Pseudomonas aeruginosa* is an opportunist Gram-negative human pathogen responsible for a variety of nosocomical infections and life-threatening diseases in immunocompromised and debilitated patients [1,2]. *P. aeruginosa* infections are notoriously difficult to eradicate, which has been attributed to the predilection of *P. aeruginosa* cells to form antibiotic-resistant biofilms, and high levels of intrinsic antibiotic resistance [1,2,3]. Indeed, multi-drug resistance *P. aeruginosa* nosocomical infections are increasingly being detected across the globe [4]. Thus the exploration of new strategies for tackling infections caused by this notorious pathogen is urgently warranted [1,2,5]. The ability of *P. aeruginosa* to cause disease is dependent upon the production of agents called ‘virulence factors’ that actively cause damage to host tissues [1,6,7,8]. The targeting of virulence factors (for example, inhibition of their production) has been identified as a potential new therapeutic approach to treating *P. aeruginosa* infections; in principle, this would attenuate the pathogenicity of the bacterium, increasing the likelihood that the host immune system can clear the infection before too much tissue damage is caused [1,7,9,10,11]. One of the many virulence factors produced by *P. aeruginosa* is pyocyanin (Figure 1) [12]. There is a large body of evidence that this low molecular weight redox-active phenazine dye is important to the pathogenesis of *P. aeruginosa* infections [1,5,12,13,14]. Unsurprisingly therefore, the inhibition of pyocyanin production has been identified as a potential antivirulence strategy against this organism [1,5,14,15]. Indeed, there has been much interest in the discovery of compounds with the ability to inhibit pyocyanin biosynthesis in recent years [1,16,17,18,19,20].

Pyocyanin production in *P. aeruginosa* is regulated by an intercellular signaling process known as quorum sensing [21,22]. Many species of bacteria use quorum sensing systems, which allows for concerted interactions between the cells comprising a population [9]. This communication process is mediated by small diffusible signaling molecules termed autoinducers [9,20,23]. In the majority of Gram-negative species, *N*-acylated-L-homoserines (AHLs) serve as the autoinducers [20,23]. These are produced by LuxI-type synthase enzymes and bind to cyctoplasmic LuxR-type receptors to initiate the expression of genes associated with bacterial group processes [1,20,23,24,25]. In general, each bacterial species responds specifically to its own unique AHL(s), and uses different LuxI-type synthases and LuxR-type receptors [23,26]. Two AHL-based quorum sensing systems are present in *P. aeruginosa*. One employs *N*-butanoyl-L-homoserine lactone (BHL, Figure 1) as the signaling molecule (generated by RhlI and detected by RhlR) and the other uses *N*-(3-oxododecanoyl)-l-homoserine lactone (OdDHL, Figure 1, generated by LasI with LasR as the cognate receptor). There is a third quorum sensing system in *P. aeruginosa* which employs a chemically distinct autoinducer (termed the *Pseudomonas* quinolone signal, PQS). The PQS system is interlinked with the two AHL-based systems, forming an intricate hierarchical quorum sensing network, with the *las* system generally regarded as standing at the apex [1,23,27,28]. The production of pyocyanin is regulated by RhlR and transcription of the *rhlR* gene itself is regulated by LasR [1,21]. Thus, inhibitors of LasR would be expected to attenuate the biosynthesis of pyocyanin [1,20,23,29,30,31]. The structure of OdDHL, the natural LasR agonist, has often been used as a template to guide the design and synthesis of abiotic LasR antagonists [1,11,16,20,23]. We recently reported the synthesis of OdDHL analogues containing non-native head groups in place of the natural homoserine lactone moiety [1]. These compounds were evaluated for their ability to inhibit the production of pyocyanin in cultures of wild type *P. aeruginosa*, with **1** (Figure 1) found to be the most potent (note that compound **1** was not screened in any LasR-based reporter systems).

Given that **1** is closely related in structure to OdDHL (which is known to interact with the LasR receptor) and the fact that pyocyanin production is generally considered to be regulated by LasR-based quorum sensing, it was postulated that **1** reduces the level of pyocyanin production by disrupting OdDHL-dependent activation of LasR [1]. Experimental evidence suggested that **1** is capable of binding to LasR and it was inferred that **1** might be an antagonist of the LasR receptor and an inhibitor of LasR-based quorum sensing in* P. aeruginosa*. This could have important implications; quorum sensing is known to regulate many additional facets of *P. aeruginosa* pathogenicity [15,32,33,34] and there is tremendous interest in finding small molecules that can disrupt AHL-mediated signaling in this organism [9,20,23]. However, our previous studies did not provide any *direct* evidence for an interaction between compound **1** and the LasR receptor, or indeed any other molecular targets. We were therefore interested in examining the molecular basis for the activity of **1** in more detail. Such information should assist in the design of next-generation agents with improved potency. Towards this end, we envisaged the design and synthesis of an affinity-based (“pull down”) chemical probe incorporating **1**. This could potentially be employed in affinity-based (“pull down”) proteomic assays in order to directly detect the biological target(s) of **1** and thus better delineate the mechanism by which it reduces the level of pycocyanin production in *P. aeruginosa* [35,36,37,38,39,40,41].

## 2. Results and Discussion

### 2.1. Probe Design

**Figure 2:**
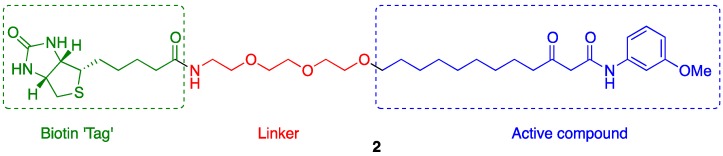
The target biotinylated affinity probe.

Typically, affinity-based probes are composed of the biologically active molecule of interest tethered *via* a chemical linker to an insoluble support [38]. Usually, the probe is then incubated with the cell lysate of the relevant organism [42]. The small molecule’s macromolecular targets can then be extracted by virtue of specific binding; washing steps are used to remove non-binding proteins, and the remaining high affinity binders can be eluted from the support, separated using polyacrylaminde gel electrophoresis and identified using various mass spectrometry techniques [35,36,37,42]. Biotin is often used as an equivalent of an insoluble support (“tag”) in affinity probes, since immobilization on streptavidin beads (either before or after incubation with the biological system) is possible by virtue of the strong non-covalent biotin-streptavidin interaction [35,37,42]. Indeed, biotinylated probes have been widely used for the identification of many small molecule biological targets [38]. An advantage of biotinylated probes over solid-phase supports in that they are often cell permeable. Thus in addition to carrying out experiments using cell lysates, it is also possible for such probes to be incubated with live cells and interact with target protein(s) in their native environment inside a living cell or organism [37]. After cell lysis the probe can be pulled out of solution with streptavidin resin, which will also pull out any bound protein(s) [37]. Based on these considerations, we targeted the synthesis of **2** (Figure 2), a biotinylated affinity probe that could potentially be used for detecting the molecular targets of the active compound **1**.

It was decided to use a polyethylene glycol (PEG)-based chain as the linker. PEG chains are commonly employed in this regard; they are usually long enough to mitigate undesired steric interactions steric hindrance between the support and small molecule-biomolecule interactions [38] and flexible enough to allow the target molecule to adopt multiple orientations in three-dimensions (and so access a favorable macromolecular binding pose). Furthermore, PEG linkers are also hydrophilic, increasing the solubility of molecules in aqueous solution [38]. A crucial consideration when preparing an affinity-based probe is where on the molecule of interest the linker should be introduced, as it is important that its’ biological activity is not affected significantly [38]. Our previous studies [1] indicated that further substitution of the aromatic ring portion would not be appropriate, as the nature of the aromatic head group was found to have a profound effect upon the ability of OdDHL-mimics of the type of **1** to inhibit pyocyanin production (with strongest inhibition associated with the *meta*-methoxy aromatic ring of **1**). There exists a subtle interplay between the structural and electronic properties of the aromatic ring group governing compound activity, meaning that the impact of further substitution could not be reliably predicted. Furthermore, evidence suggests that the natural 3-oxo-dodecanoyl tail group of OdDHL is important for the inhibition of pyocyanin production by compounds which mimic the structure of AHLs. We therefore decided to retain the dicarbonyl unit and the nine-carbon alkyl chain, and attach the linker at the end of the alkyl chain.

### 2.2. Probe Synthesis

**Scheme 1:**
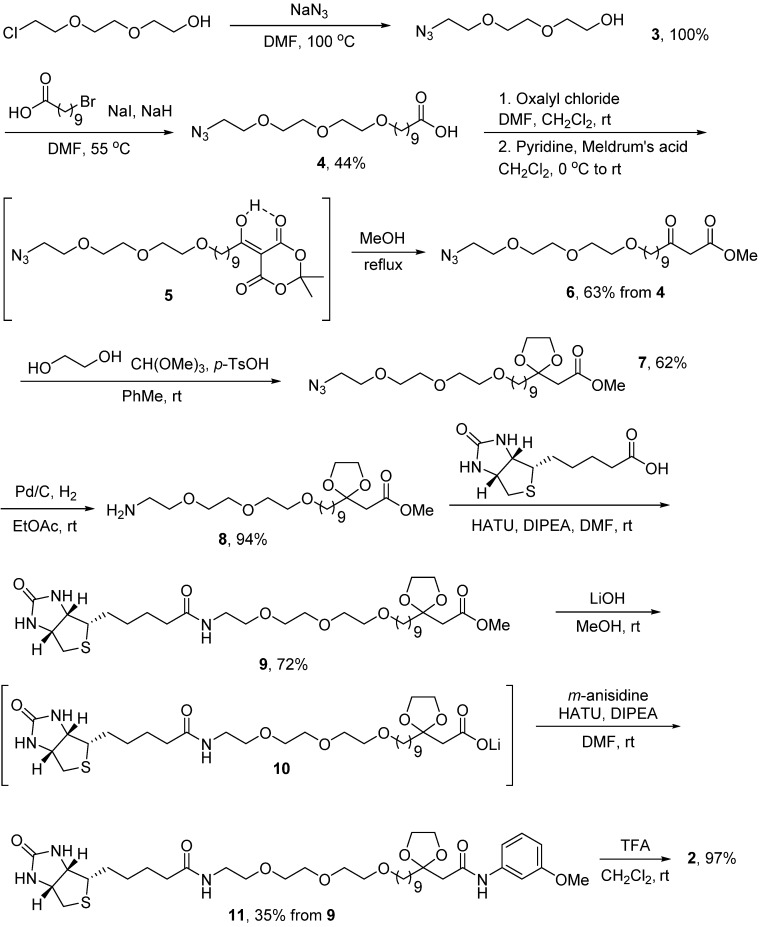
The synthesis of biotin-tagged affinity probe **2**. rt = room temperature.

The synthesis of compound **2** (Scheme 1) began with the reaction of 2-(2-(2-chloroethoxy)ethoxy)-ethanol and sodium azide, which furnished azide **3** in quantitative yield. The desired nine-carbon alkyl chain was installed by reaction deprotonation of the hydroxyl group and treatment with 10-bromodecanoic acid.

The resulting acid **4** was converted to the corresponding acid chloride and reacted with Meldrum’s acid to generate adduct **5**. Subsequent treatment of this crude material with methanol yielded β-ketoester **6**. Acetal protection to form **7** was followed by reduction of the azide group to yield **8**. Subsequent HATU-mediated coupling with D-biotin proceeded smoothly to generate the protected ester **9**. Hydrolysis using lithium hydroxide provided the carboxylate **10**. HATU-mediated coupling of **10** with *m*-anisidine furnished the protected precursor **11** in reasonable yield. Finally, acid-catalyzed deprotection provided the desired compound **2**.

Typical affinity-based pull down assays involve the use of one-dimensional (1D) gel electrophoresis to separate proteins binding to the bioactive molecule under investigation. However, this method can suffer from a lack of sensitivity. We have previously proposed a strategy to address this issue based around combining biotin-mediated affinity capture from cell lysates with 2D difference gel electrophoresis (DIGE) [42]. This method of electrophoresis should allow a greater sensitivity compared to 1D techniques and thus facilitate the identification of weak binding-protein targets [42]. Conceivably, such an approach could be used with the affinity probe 2 in order to elucidate molecular targets for the anti-pyocyanin compound **1**.

## 3. Experimental

### 3.1. General

Reactions were performed using oven-dried glassware under an atmosphere of nitrogen with anhydrous, freshly distilled solvents unless otherwise stated. Dichloromethane, ethyl acetate, methanol, *n*-hexane, acetonitrile and toluene were distilled from calcium hydride. Diethyl ether was distilled over a mixture of lithium aluminium hydride and calcium hydride. Petroleum ether was distilled before use and refers to the fraction between 40–60 °C. All other reagents were used as obtained from commercial sources. Room temperature (rt) refers to ambient temperature. Temperatures of 0 °C were maintained using an ice-water bath. Reaction times are given either in hours (h) or minutes (min) or overnight (approximately 12 h) Yields refer to chromatographically and spectroscopically pure compounds unless otherwise stated. All flash chromatography was carried out using slurry-packed Merck 9325 Keiselgel 60 silica gel. Where possible, reactions were monitored by thin layer chromatography (TLC) performed on commercially prepared glass plates pre-coated with Merck silica gel 60 F254 or aluminium oxide 60 F254. Visualisation was by the quenching of UV fluorescence (ν*_max_* = 254 nm) or by staining with potassium permanganate. Infrared spectra were recorded neat or as a solution in the designated solvent on a Perkin-Elmer Spectrum One spectrometer with internal referencing. Selected absorption maxima (ν*_max_*) are reported in wavenumbers (cm^−1^). Proton magnetic resonance spectra were recorded using an internal deuterium lock at ambient probe temperatures (unless otherwise stated) on the following instruments: Bruker DPX-400 (400 MHz), Bruker Avance 400 QNP (400 MHz) Bruker Avance 500 BB ATM (500 MHz) and Bruker Avance 500 Cryo Ultrashield (500 MHz). Chemical shifts (δ_H_) are quoted in ppm, to the nearest 0.01 ppm, and are referenced to the residual non-deuterated solvent peak. Coupling constants (*J*) are reported in Hertz. Data are reported as follows: chemical shift, integration, multiplicity [br, broad; s, singlet; d, doublet; t, triplet; q, quartet; quint, quintet; sext, sextet; sept, septet; m, multiplet; or as a combination of these (e.g., dd, dt, *etc.*)], coupling constant(s) and assignment. Proton assignments were determined either on the basis of unambiguous chemical shift or coupling pattern, by patterns observed in 2D experiments (^1^H-^1^H COSY, HMBC and HMQC) or by analogy to fully interpreted spectra for related compounds. Carbon magnetic resonance spectra were recorded by broadband proton spin decoupling at ambient probe temperatures (unless otherwise stated) using an internal deuterium lock on the following instruments: Bruker DPX-400 (100 MHz), Bruker Avance 400 QNP (100 MHz) and Bruker Avance 500 BB ATM (125 MHz) and Bruker Avance 500 Cryo Ultrashield (125 MHz). Chemical shifts (δ_C_) are quoted in ppm, to the nearest 0.1 ppm, and are referenced to the residual non-deuterated solvent peak. Assignments were supported by DEPT editing and determined either on the basis of unambiguous chemical shift or coupling pattern, by patterns observed in 2D experiments (HMBC and HMQC) or by analogy to fully interpreted spectra for related compounds. The ionisation technique used was electron ionisation (EI). High resolution mass spectroscopy measurements were recorded in-house using a Waters LCT Premier Mass Spectrometer or a Micromass Quadrapole-Time of Flight (Q-ToF) spectrometer. Mass values are reported within the error limits of ±5 ppm mass units. The ionisation technique used was electrospray ionization (ESI). Optical rotations were measured in MeOH on a Perkin Elmer 343 Polarimeter; [α]_D_ values are reported in 10^−1^ degrees cm^2^ g^−1^ at 589 nm.

### 3.2. Experimental Procedures

*2-(2-(2-Azidoethoxy)ethoxy)ethanol* (**3**). To a solution of NaN_3_ (2.06 g, 32.7 mmol, 1.4 eq.) in DMF (10 mL) was added 2-(2-(2-chloroethoxy)ethoxy)ethanol (4 g, 23.8 mmol 1 eq.). The reaction mixture was stirred at 100 °C for 5 h and evaporated to dryness. The resulting residue was partitioned between CH_2_Cl_2_ (20 mL) and water (20 mL). The aqueous phase was extracted twice more with CH_2_Cl_2_ (2 × 50 mL), the organic layers combined, dried over MgSO_4_ and evaporated to dryness to give **3** as a pale yellow oil (4.21 g, 24 mmol, 100%) which was used without further purification. R*_f_* 0.16 (70% EtOAc/bp 40–60 petroleum ether); ν_max_ (neat)/cm^−1^: 3400 (broad, O-H), 2869 (C-H), 2099 (N_3_); ^1^H-NMR (400 MHz, CDCl_3_) δ_H_ 3.73 (2H, t, *J* = 5.5 Hz, CH_2_OH), 3.69-3.65 (6H, m, 3 × CH_2_O), 3.61 (2H, t, *J* = 4.6 Hz, CH_2_O), 3.30 (2H, t, *J* = 5.0 Hz, CH_2_N_3_), 2.36 (1H, t, *J* = 6.2 Hz, CH_2_OH); ^13^C-NMR (101 MHz, CDCl_3_) δ_C_ 72.6 (CH_2_O), 70.7 (CH_2_O), 70.5 (CH_2_O), 70.2 (CH_2_O), 61.9 (CH_2_OH), 50.8 (CH_2_N_3_).

*10-(2-(2-(2-Azidoethoxy)ethoxy)ethoxy)decanoic acid* (**4**). A solution of bromodecanoic acid (909 mg, 5.2 mmol, 1 eq.), NaI (78 mg, 0.52 mmol, 0.1 eq.) and compound **3** (1.3 g, 5.2 mmol, 1 eq.) in dry DMF (10 mL) was cooled to 0 °C and NaH (60% dispersion in mineral oil, 520 mg, 13 mmol, 2.5 eq.) was added piecewise. After 20 min the reaction mixture was heated to 55 °C and stirred overnight forming an orange gel. The solvent was removed under reduced pressure and the residue was partitioned between HCl_aq_ (100 mL) and Et_2_O (100 mL). The aqueous phase was extracted with Et_2_O (2 × 100 mL), the combined organic layers dried over MgSO_4_ and evaporated to dryness. The resulting residue was purified by column chromatography (stepwise gradient, 0%–40% Et_2_O/bp 40–60 petroleum ether with 1% acetic acid) to yield **4** (0.789 g, 1.78 mmol, 44%) as a colourless oil. R*_f_*: 0.34 (40% EtOAc/bp 40–60 petroleum ether, 1% acetic acid); ν_max_ (neat)/cm^−1^: 3400 (O-H), 2927 (C-H), 2856 (C-H), 2100 (N_3_), 1708 (C=O); ^1^H-NMR (500 MHz, CDCl_3_) δ_H_ 3.70–3.64 (8H, m, 4 × OCH_2_), 3.59 (2H, app dd, *J* = 6.0, 3.8 Hz, OCH_2_), 3.45 (2H, t, *J* = 6.8 Hz, OCH_2_), 3.39 (2H, t, *J* = 5.1 Hz, CH_2_N_3_), 2.34 (2H, t, *J* = 7.5 Hz, HOOCCH_2_), 1.80–1.50 (4H, m, HOOCCH_2_CH_2_ and CH_2_CH_2_CH_2_O), 1.31 (10H, d, *J* = 18.8 Hz, 5 × CH_2_); ^13^C-NMR (126 MHz, CDCl_3_) δ_C_ 178.3 (HOOCCH_2_) 71.7 (CH_2_O), 70.9 (CH_2_O), 70.9 (CH_2_O), 70.8 (CH_2_O), 70.2 (CH_2_O), 70.2(CH_2_O), 50.9 (CH_2_N_3_), 33.9 (HOOCCH_2_), 29.7 (CH_2_), 29.5 (CH_2_), 29.4 (CH_2_), 29.2 (CH_2_), 29.1 (CH_2_), 26.2 (CH_2_), 24.8 (HOOCCH_2_CH_2_); HRMS (ESI^+^) *m/z* found [M+H]^+^ 346.2354, [C_16_H_32_N_3_O_5_]^+^ calculated 345.2342.

*Methyl 12-(2-(2-(2-azidoethoxy)ethoxy)ethoxy)-3-oxododecanoate* (**6**) To a solution of compound **4** (242 mg, 0.70 mmol, 1 eq) in dry CH_2_Cl_2_ (1.5 mL) at rt was added oxalyl chloride (100 μL, 1.19 mmol, 1.7 eq.) and DMF (10 μL). After stirring at rt for 1 h TLC indicated complete turnover (small samples of the reaction mixture were quenched with MeOH), the solvent was removed under reduced pressure and the acid chloride was used without further purification. A solution of Meldrum’s acid (101 mg, 0.7 mmol, 1 eq.) in dry CH_2_Cl_2_ (1.6 mL) was cooled to 0 °C and pyridine (114 μL, 1.4 mmol, 2 eq.) was added dropwise over 20 min. The acid chloride in CH_2_Cl_2_ (1 mL) was then added and the mixture was stirred at 0 °C for a further 2 h. The reaction mixture was allowed to warm to rt diluted with CH_2_Cl_2_ (10 mL) and poured into ice HCl (2N, 15 mL). The organic layer was washed with NaCl_aq_ (25 mL), dried over MgSO_4_ and evaporated to dryness. The resultant crude product material was dissolved in methanol (2.5 mL) and heated to reflux with stirring for 5 h. The solvent was removed under reduced pressure and the resulting residue was purified by column chromatography (40% Et_2_O/bp 40–60 petroleum ether) to yield **6** (176 mg, 0.416 mmol, 63%) as a pale yellow oil. R*_f_* 0.22 (40% Et_2_O/bp 40–60 petroleum ether); ν_max_ (neat)/cm^−1^: 2925 (C-H), 2857 (C-H), 2101 (N_3_), 1747 (C=O), 1717 (C=O); ^1^H-NMR (400 MHz, CDCl_3_) δ_H_ 3.74 (3H, s, CH_3_), 3.70–3.62 (8H, m, 4 × OCH_2_), 3.60–3.55 (2H, m, OCH_2_CH_2_N_3_), 3.45 (2H, t, *J* = 6.77 Hz, CH_2_CH_2_CH_2_O), 3.44 (2H, s, COCH_2_CO) 3.39 (2H, t, *J* = 5.10 Hz, CH_2_N_3_), 2.52 (2H, t, *J* = 7.37 Hz, COCH_2_CH_2_), 1.65–1.51 (4H, app. m (also H_2_O), COCH_2_CH_2_CH_2_ and CH_2_CH_2_CH_2_O), 1.39–1.23 (10H, m, 5 × CH_2_); ^13^C-NMR (101 MHz, CDCl_3_) δ_C_ 203.0 (CH_2_COCH_2_), 167.8 (H_3_CCO), 71.6 (CH_2_CH_2_CH_2_O), 70.9 (CH_2_O), 70.9 (CH_2_O), 70.8 (CH_2_O), 70.2 (CH_2_O), 70.2 (CH_2_O), 52.5 (H_3_C), 50.8 (COCH_2_CO), 49.2 (CH_2_N_3_), 43.2 (COCH_2_CH_2_), 29.7 (CH_2_), 29.5 (CH_2_), 29.5 (CH_2_), 29.4 (CH_2_), 29.1 (CH_2_), 26.2 (CH_2_), 23.6 (COCH_2_CH_2_CH_2_); HRMS (ESI^+^) *m/z* found [M+H]^+^ 424.2411 [C_19_H_35_N_3_O_6_^23^Na_1_]^+^ calculated 424.2418.

*Methyl 2-(2-(9-(2-(2-(2-azidoethoxy)ethoxy)ethoxy)nonyl)-1,3-dioxolan-2-yl)acetate* (**7**). To a solution of the β keto ester **6** (100 mg, 0.24 mmol, 1 eq.) in toulene (5 mL) at rt was added tosic acid (12.2 mg, 0.1 eq.), trimethylorthoformate (0.21 mL, 1.2 mmol, 5 eq.) and ethylene glycol (0.119 mL, 2.13 mmol, 8.9 eq.) sequentially with stirring. The reaction was left to stir overnight at rt. The toluene was removed under reduced pressure and the resulting reside was disolved in CH_2_Cl_2_ (10 mL), washed with saturated aqueous sodium bicarbonate solution (3 × 10 mL), dried over MgSO_4_ and evaporated to dryness. The resulting crude product was purrified by column chromatography (stepwise gradient, 10%–40% Et_2_O/bp 40-60 petroleum ether) to yield 7 (69 mg, 0.148 mmol, 62%) as a colourless oil. R*_f_*: 0.42 (70% Et_2_O/bp 40-60 petroleum ether); ν_max_ (neat)/cm^−1^: 2924 (C-H), 2855 (C-H), 2103 (N_3_), 1738 (C=O); ^1^H-NMR (500 MHz, CDCl_3_) δ_H_ 4.01–3.94 (4H, m, OCH_2_CH_2_O (actetal)), 3.69 (3H, s, OCH_3_), 3.68–3.65 (8H, m, 4 × CH_2_O), 3.58 (2H, app dd, *J* = 3.60, 5.90 Hz, OCH_2_), 3.45 (2H, t, *J* = 6.82 Hz, CH_2_CH_2_CH_2_O), 3.39 (2H, t, *J* = 5.12 Hz, CH_2_N_3_), 2.66 (2H, s, CH_3_OOCCH_2_), 1.85–1.74 (2 H, app. M, CCH_2_), 1.55 (2H, dt, *J* = 22.0, 11.2 Hz, CH_2_CH_2_CH_2_O), 1.45–1.18 (12H, m, 6 × CH_2_); ^13^C (126 MHz, CDCl_3_) δ_C_ 170.2 (CH_3_OOC), 109.6 (CH_3_OOCCH_2_CO), 71.7 (CH_2_CH_2_CH_2_O), 70.9 (OCH_2_), 70.9 (OCH_2_), 70.8 (OCH_2_), 70.2 (OCH_2_), 70.2 (OCH_2_), 65.3 (OCH_2_CH_2_O acetal), 51.9 (CH_3_O), 50.9 (CH_2_N_3_), 42.6 (CH_3_OOCCH_2_), 37.9 (CCH_2_), 29.8 (CH_2_), 29.8 (CH_2_), 29.6 (CH_2_), 29.6 (CH_2_), 29.6 (CH_2_), 26.2 (CH_2_), 23.6 (CCH_2_CH_2_). HRMS (ESI^+^) *m/z* found [M+H]^+^ 468.2675 [C_21_H_39_N_3_O_7_^23^Na_1_]^+^ calculated 468.2680.

*Methyl 2-(2-(9-(2-(2-(2-aminoethoxy)ethoxy)ethoxy)nonyl)-1,3-dioxolan-2-yl)acetate* (**8**) To a solution of compound **7** (62.0 mg, 0.133 mmol, 1 eq.) in degassed EtOAc (2 mL) under an inert N_2_ atmosphere was added Pd (5% on carbon, 7.8 mg, 0.0037 mmol, 0.028 eq.). The reaction vessel was then purged with H_2_ before being stirred under an atmosphere of H_2_ for overnight at rt. The reaction mixture was filtered through celite (washed with EtOAc) and evaporated to dryness to give compound **8** (52.2 mg, 0.124 mmol, 94%) as a yellow oil which was used without further purification. ν_max_ (neat)/cm^−1^: 3373 (N-H), 2925 (C-H), 2855 (C-H), 1738 (C=O), 1739 (C=O), 1672; ^1^H-NMR (500 MHz, CDCl_3_) δ_H_ 4.02–3.93 (4H, m, OCH_2_CH_2_O (acetal)), 3.69 (3H, s, OCH_3_), 3.68–3.53 (10H, m, 5 × CH_2_O), 3.45 (2H, t, *J* = 6.84 Hz, CH_2_CH_2_CH_2_O), 2.91 (2H, dt, *J* = 10.57, 5.16 Hz, CH_2_NH_2_), 2.66 (2H, s, CH_3_OOCCH_2_), 2.36 (2H, br s, CH_2_NH_2_), 1.81–1.72 (2 H, app. m, CCH_2_), 1.55 (2H, m, CH_2_CH_2_CH_2_O), 1.41–1.23 (12H, m, 6 × CH_2_); ^13^C (126 MHz, CDCl_3_) δ_C_ 170.2 (CH_3_OOC), 109.6 (CH_3_OOCH_2_C), 72.4 (OCH_2_CH_2_NH_2_), 71.7 (CH_2_CH_2_CH_2_O), 70.8 (OCH_2_), 70.7 (OCH_2_), 70.4 (OCH_2_), 70.2 (OCH_2_), 65.3 (OCH_2_CH_2_O acetal), 51.9 (CH_3_O), 42.6 (CH_3_OOCCH_2_), 41.5 (CH_2_NH_2_), 37.9 (CCH_2_), 29.8 (CH_2_), 29.7 (CH_2_), 29.6 (CH_2_), 29.6 (CH_2_), 29.6 (CH_2_), 26.2 (CH_2_), 23.6 (CCH_2_CH_2_); HRMS (ESI^+^) *m/z* found [M+H]^+^ 420.2947 [C_21_H_42_N_3_O_7_]^+^ calculated 420.2956.

*Methyl 2-(2-(5-oxo-1-((3aS,4S,6aR)-2-oxohexahydro-1H-thieno[3,4-d]imidazol-4-yl)-9,12,15-trioxa-6-azatetracosan-24-yl)-1,3-dioxolan-2-yl)acetate* (**9**) To a solution of D-biotin (31.2 mg, 0.128 mmol, 1.16 eq.) in DIPEA (1.77 mL) at rt was added HATU (41.7 mg, 0.110 mmol, 1 eq.) with stirring. The solution was stirred for a further 15 min before the addition of compound **8** (46 mg, 0.110 mmol, 1 eq.). This was stirred at rt overnight. The reaction was quenched by the addition of MeOH (1 mL), the solvent removed under reduced pressure and the resulting residue purified by column chromatography (stepwise gradient, 1%–10% MeOH/CH_2_Cl_2_) to give **9** (50.9 mg, 0.079 mmol, 72%) as a sticky pink syrup. R*_f_*: 0.21 (5% MeOH/CH_2_Cl_2_); ν_max_ (neat)/cm^−1^: 3294 (N-H amide), 2926 (C-H), 2854 (C-H), 1739 (C=O), 1700 (C=O), 1645 (C=O); ^1^H-NMR (500 MHz, CDCl_3_) δ_H_ 6.56 (1H, br s, OCH_2_CH_2_NHCO), 5.99 (1H, br s, NHCONH), 5.17 (1H, br s, NHCONH), 4.49–4.43 (1H, m, CONHCHCH_2_S), 4.30–4.23 (1H, m, NHCHCHS), 3.94–3.86 (4H, m, OCH_2_CH_2_O acetal), 3.62 (3H, s, CH_3_O), 3.59–3.54 (6H, m, 3 × OCH_2_), 3.53–3.47 (4H, m, OCH_2_ and OCH_2_CH_2_NH), 3.37 (4H, m, CH_2_CH_2_CH_2_O and OCH_2_CH_2_NH), 3.09 (1H, dd, *J* = 11.10, 6.89 Hz, NHCHCHS), 2.85 (1H, dd, *J* = 12.75, 4.47 Hz, CONHCHCHHS), 2.85 (1H, d, *J* = 12.77 Hz, CONHCHCHHS), 2.59 (2H, s, CH_3_OOCCH_2_), 2.22–2.11 (2H, m, NHCOCH_2_CH_2_CH_2_), 1.74–1.68 (2H, app. m, CCH_2_), 1.67–1.54 (4H, m, NHCOCH_2_CH_2_CH_2_), 1.50 (2H, dt, *J* = 13.94, 6.83 Hz, CH_2_CH_2_CH_2_CH_2_O), 1.38 (2H, dt, *J* = 14.93, 7.52 Hz, NHCOCH_2_CH_2_CH_2_), 1.34–1.27 (2H, m, CCH_2_CH_2_), 1.27–1.16 (10H, m, 5 × CH_2_); ^13^C-NMR (126 MHz, CDCl_3_) δ_C_ 173.4 (CH_2_NHCOCH_2_), 170.2 (CH_3_OOC), 163.6 (NHCONH), 109.6 (CH_2_CCH_2_), 71.7 (CH_2_CH_2_CH_2_O), 70.7, (OCH_2_), 70.6 (OCH_2_), 70.3 (OCH_2_), 70.2 (OCH_2_), 70.0 (OCH_2_), 65.3 (OCH_2_CH_2_O acetal), 62.0 (NHCHCHS), 60.4 (NHCHCH_2_S), 55.4 (NHCHCHS), 51.9 (CH_3_O), 42.6 (COCH_2_C), 40.7 (NHCHCH_2_S), 39.4 (OCH_2_CH_2_NH), 37.9 (COCH_2_CCH_2_), 35.9 (NHCOCH_2_CH_2_CH_2_), 29.8 (CH_2_), 29.8 (CH_2_), 29.7 (CH_2_), 29.7 (CH_2_), 29.6 (CH_2_), 28.2 (CH_2_), 28.1 (CH_2_), 26.2 (CH_2_), 25.6 (CH_2_), 23.6 (CCH_2_CH_2_); HRMS (ESI^+^) *m/z* found [M+H]^+^ 646.3278 [C_31_H_56_N_3_O_9_S]^+^ calculated 646.3732; ${\left[\alpha \right]}_{D}^{26.7}$ +28 (c = 0.917 mmol in MeOH).

*Lithium 2-(2-(5-oxo-1-((3aS,4S,6aR)-2-oxohexahydro-1H-thieno[3,4-d]imidazol-4-yl)-9,12,15-trioxa-6-azatetracosan-24-yl)-1,3-dioxolan-2-yl)acetate* (**10**) Compound **9** (48 mg, 0.074 mmol, 1 eq.) was dissolved in 66% aqueous MeOH (5 mL), lithium hydroxide monohydrate (21 mg, 0.5 mmol, 6.8 eq.) was added and the reaction stirred overnight at rt. The solvent was removed under reduced pressure to give a mixture of compound **10** and LiOH (69 mg) which was used without further purification. ν_max_ (neat)/cm^−1^: 3277 (N-H amide), 2924 (C-H), 2854 (C-H), 1686 (C=O), 1593 (C=O); ^1^H-NMR (500 MHz, CD_3_OD) δ_H_ 4.46 (1H, ddd, *J* = 7.85, 4.94, 0.73 Hz, NHCHCH_2_S), 4.27 (1H, dd, *J* = 7.88, 4.47 Hz, NHCHCHS)), 4.03–3.80 (4H, m, OCH_2_CH_2_O acetal), 3.65–3.57 (6H, m, 3 × OCH_2_), 3.55 (2H, m, OCH_2_), 3.51 (2H, t, *J* = 5.46 Hz, OCH_2_CH_2_NH), 3.44 (2H, t, *J* = 6.65 Hz, CH_2_CH_2_CH_2_O), 3.33 (2H, t, *J* = 5.33 Hz, OCH_2_CH_2_NH), 3.20–3.14 (1H, m, NHCHCHS), 2.90 (1H, dd, *J* = 12.75, 5.00 Hz, CONHCHCHHS), 2.67 (1H, d, *J* = 12.72 Hz, CONHCHCHHS), 2.43 (2H, s, CH_3_OOCCH_2_), 2.19 (2H, t, *J* = 7.40 Hz, NHCOCH_2_CH_2_CH_2_), 1.82–1.76 (2H, app. m, CCH_2_), 1.75–1.49 (6H, m, 3 × CH_2_), 1.45–1.34 (m, 4H, 2 × CH_2_), 1.34–1.19 (10H, m, 5 × CH_2_); ^13^C-NMR (126 MHz, CD_3_OD) δ_C_ 178.3 (CO), 176.2 (CO), 166.1 (NHCONH), 111.6 (CH_2_CCH_2_), 72.4 (CH_2_CH_2_CH_2_O), 71.6 (OCH_2_), 71.6 (OCH_2_), 71.3 (OCH_2_), 71.1 (OCH_2_), 70.6 (OCH_2_), 65.8 (OCH_2_CH_2_O acetal), 63.4 (NHCHCHS), 61.6 (NHCHCH_2_S), 57.0 (NHCHCHS), 47.0 (COCH_2_CO), 41.1 (NHCHCH_2_S), 40.4 (OCH_2_CH_2_NH), 38.7 (COCH_2_CCH_2_), 36.7 (NHCOCH_2_CH_2_CH_2_), 31.0 (CH_2_), 30.7 (CH_2_), 30.7 (CH_2_), 30.7 (CH_2_), 30.6 (CH_2_), 29.8 (CH_2_), 29.5 (CH_2_), 27.2 (CH_2_), 26.9 (CH_2_), 24.6 (CCH_2_CH_2_); HRMS (ESI^+^) *m/z* found [M+H]^+^ 632.3584 [C_30_H_59_N_3_O_9_S]^+^ calculated 632.3575.

*N-(2-(2-(2-((9-(2-(2-((3-Methoxyphenyl)amino)-2-oxoethyl)-1,3-dioxolan-2-yl)nonyl)oxy)ethoxy)ethoxy)ethyl)-5-((3aS,4S,6aR)-2-oxohexahydro-1H-thieno[3,4-d]imidazol-4-yl)pentanamide* (**11**) To a solution of *m*-anisidine (16 µL, 0.142 mmol, 2 eq.) in a solution of DIPEA (12.2 µL) and DMF (1 mL) at rt was added HATU (27 mg, 0.071 mmol, 1 eq.) with stirring. The solution was stirred for a further 15 min before the addition of the crude compound **10** (65.3 mg, ≈0.071 mmol, 1 eq.). This was stirred at rt overnight. The reaction was quenched by the addition of MeOH (1 mL), the solvent removed under reduced pressure and the resulting residue purified by column chromatography (stepwise gradient, 1%–7% MeOH/CH_2_Cl_2_) to give **11** (18.4 mg, 0.025 mmol, 35% over two steps) as a sticky pale orange residue. R*_f_*: 0.5 (10% MeOH/CH_2_Cl_2_); ν_max_ (neat)/cm^−1^: 3647 (N-H amide) 3437 (N-H amide) 2926 (C-H), 2853 (C-H), 1662 (C=O), 1609 (C=O), 1544 (C=O); ^1^H-NMR (400 MHz, CD_3_OD) δ_H_ 9.53 (1H, br s, CNHCO), 7.30 (1H, dd, *J* = 4.27, 2.09 Hz, CH_3_OCCHCNH), 7.23 (1H, t, *J* = 8.15 Hz, CCHCHCHC), 7.08 (1H, ddd, *J* = 8.07, 1.76, 0.76 Hz, CCHCHCHC), 6.70 (1H, ddd, *J* = 8.28, 2.48, 0.73 Hz, CCHCHCHC), 4.52 (1H, dd, *J* = 7.90, 4.33 Hz, NHCHCH_2_S), 4.33 (1H, dd, *J* = 7.90, 4.40 Hz, NHCHCHS), 4.14–3.88 (4H, m, OCH_2_CH_2_O acetal), 3.81 (3H, s, CH_3_O), 3.71–3.59 (8H, m, 4 × OCH_2_), 3.57 (2H, t, *J* = 5.45 Hz, OCH_2_CH_2_NH), 3.50 (2H, t, *J* = 6.63 Hz, CH_2_CH_2_CH_2_O), 3.39 (2H, t, *J* = 5.41 Hz, OCH_2_CH_2_NH), 3.22 (1H, dt, *J* = 27.40, 11.44 Hz, NHCHCHS), 2.96 (1H, dt, *J* = 12.74, 4.17 Hz, CONHCHCHHS), 2.73 (1H, d, *J* = 13.8 Hz, CONHCHCHHS), 2.71 (2H, s, NHCOCH_2_CO), 2.25 (2H, t, *J* = 7.38 Hz, NHCOCH_2_CH_2_CH_2_), 1.84–1.54 (8H, m, 4 × CH_2_), 1.54-1.42 (4H, m, 2 × CH_2_), 1.43–1.28 (10H, m, 5 × CH_2_); ^13^C-NMR (126 MHz, CD_3_OD) δ_C_ 176.1 (CO), 170.7 (CO), 167.6 (NHCONH), 166.1 (CH_3_OC), 140.9 (CHCNH), 130.5 (CH-Ar), 117.0 (COCH_2_C), 113.4 (CH-Ar), 110.7 (CH-Ar), 107.1 (CH-Ar), 72.4 (CH_2_CH_2_CH_2_O), 71.6 (OCH_2_), 71.6 (OCH_2_), 71.3 (OCH_2_), 71.1 (OCH_2_), 70.6 (OCH_2_), 64.3 (OCH_2_CH_2_O acetal), 63.4 (NHCHCHS), 61.7 (NHCHCH_2_S), 57.0 (NHCHCHS), 55.7 (CH_3_O), 43.9 (NCOCH_2_CO), 41.0 (CONHCHCH_2_S), 40.4 (OCH_2_CH_2_NH), 39.1 (COCH_2_CCH_2_), 36.7 (NHCOCH_2_CH_2_CH_2_), 30.7 (CH_2_), 30.5 (CH_2_), 30.5 (CH_2_), 30.5 (CH_2_), 30.4 (CH_2_), 29.8 (CH_2_), 29.5 (CH_2_), 27.2 (CH_2_), 26.9 (CH_2_), 24.5 (CCH_2_CH_2_); HRMS (ESI^+^) *m/z* found [M+H]^+^ 737.4138 [C_37_H_61_N_4_O_9_S]^+^ calculated 737.4154; ${\left[\alpha \right]}_{D}^{26.7}$ +27 (c = 2.183 mmol in MeOH).

*N-(3-Methoxyphenyl)-3-oxo-12-(2-(2-(2-(5-((3aS,4S,6aR)-2-oxohexahydro-1H-thieno[3,4-d]imidazol-4-yl)pentanamido)ethoxy)ethoxy)ethoxy)dodecanamide* (**2**) Compound **11** (11.0 mg, 0.015 mmol, 1 eq.) was dissolved in CH_2_Cl_2_ (0.5 mL) with TFA (160 µL, 2.09 mmol, 139 eq.) in a flask open to the air and stirred for 3 h at rt. The solvent was removed under reduced pressure and the resulting residue purified by column chromatography (stepwise gradient, 1%–7% MeOH/CH_2_Cl_2_) to yield the final compound **2** (10.1 mg, 0.146 mmol, 97%) as a colourless sticky residue. R*_f_*: 0.51 (10% MeOH/CH_2_Cl_2_); ν_max_ (neat)/cm^−1^: 3641 (N-H amide) 3294 (N-H amide) 2926 (C-H), 2853 (C-H), 1682 (C=O), 1629 (C=O), 16411 (C=O), 1544 (C=O); ^1^H-NMR (500 MHz, CD_3_OD) δ_H_ 7.30 (1H, t, *J* = 2.20 Hz, CH_3_OCHCNH), 7.22 (1H, t, *J* = 8.16 Hz, CCHCHCHC), 7.07 (1H, ddd, *J* = 8.04, 1.91, 0.85 Hz, CCHCHCHC), 6.70 (1H, ddd, *J* = 8.27, 2.49, 0.79 Hz, CCHCHCHC), 4.54–4.46 (1H, m, CONHCHCH_2_S), 4.32 (1H, dd, *J* = 7.86, 4.50 Hz, NHCHCHS), 3.80 (3H, s, CH_3_O), 3.70–3.58 (8H, m, 4 × CH_2_O), 3.56 (2H, t, *J* = 5.5 Hz, OCH_2_CH_2_NH_2_), 3.52–3.44 (2H, m,CH_2_CH_2_CH_2_O), 3.42–3.36 (m, 4H, OCH_2_CH_2_NH and COCH_2_CO), 3.24–3.16 (1H, m, NHCHCHS), 2.94 (1H, dt, *J* = 12.76, 4.97 Hz, CONHCHCHHS), 2.72 (1H, d, *J* = 12.72 Hz, CONHCHCHHS), 2.63 (2H, t, *J* = 7.28 Hz, COCH_2_COCH_2_), 2.24 (2H, t, *J* = 7.35 Hz, NHCOCH_2_CH_2_), 1.82–1.52 (8H, m, 4 × CH_2_), 1.53–1.10 (14H, m, 7 × CH_2_); ^13^C-NMR (126 MHz, CD_3_OD) δ_C_ 206.8 (NHCOCH_2_CO), 176.1 (CNHCOCH_2_CO) 167.6 (OCH_2_CH_2_NHCO), 166.1 (NHCONH), 161.6 (CH_3_OC), 140.7 (CHCNH), 130.6 (CH Ar), 113.31 (CH Ar), 110.9 (CH Ar), 107.0 (CH Ar), 72.4 (CH_2_CH_2_CH_2_O), 71.6 (OCH_2_), 71.6 (OCH_2_), 71.3 (OCH_2_), 71.1 (OCH_2_), 70.6 (OCH_2_), 63.4 (NHCHCHS), 61.6 (NHCHCH_2_S), 57.0 (NHCHCHS), 55.7 (CH_3_O), 49.9 (COCH_2_CO) 43.9 (COCH_2_COCH_2_), 41.5 (CONHCHCH_2_S), 40.4 (OCH_2_CH_2_NH), 36.7 (NHCOCH_2_CH_2_CH_2_) 30.7 (CH_2_), 30.5 (CH_2_), 30.5 (CH_2_), 30.4 (CH_2_), 30.10 (CH_2_), 29.8 (CH_2_), 29.5 (CH_2_), 27.2 (CH_2_), 26.9 (CH_2_), 24.5 (COCH_2_CH_2_); HRMS (ESI^+^) *m/z* found [M+H]^+^ 693.11 [C_35_H_57_N_4_O_8_S]^+^ calculated 693.3892; ${\left[\alpha \right]}_{D}^{26.8}$ +28 (c = 2.183 mmol in MeOH).

## 4. Conclusions

We have reported the efficient synthesis of **2**, a biotin-tagged derivative of a potent inhibitor **1** of the production of the virulence factor pyocyanin in the pathogenic bacterium *P. aeruginosa*. Compound **2** could potentially be used as a chemical probe in affinity “pull-down” assays in order to identify the biological target(s) of **1** and thus help to elucidate the mechanism by which it exerts this biological effect. Such information should prove useful in the design of more potent compounds. If **2** is found to bind to LasR, this would strongly suggest that **1** is a direct antagonist of quorum sensing in *P. aeruginosa* and, as such, it would represent a valuable molecular tool for the study and manipulation of this signaling pathway. The binding studies are currently in progress and the results of these investigations will be reported in due course [43].
